# The will of young minors in the terminal stage of sickness: A case report

**DOI:** 10.1515/med-2020-0152

**Published:** 2020-06-08

**Authors:** Piergiorgio Fedeli, Sergio Giorgetti, Nunzia Cannovo

**Affiliations:** School of Law, Legal Medicine Section, University of Camerino (MC), Camerino, Italy; Hospital of San Severino Marche, AV3- Asur Marche, Macerata, Italy; Ethics Committee of Federico II University, Naples, Italy

**Keywords:** neuroblastoma, palliative sedation, informed consent, children

## Abstract

**Introduction:**

In Italy, both parents have parental responsibility, so they have the power to give or withhold consent to medical procedures on their children.

**Methods:**

The present work reports the case of a 5-year-old boy diagnosed with neuroblastoma in the right adrenal loggia, who underwent several chemotherapy treatments that prolonged his life until the age of 10. Informed consent for treatments was requested exclusively of the parents, without taking into consideration the minor’s will, not even when he asked for increased pain relief medication instead of other palliative treatments.

**Results:**

The authors thought it interesting to examine the case in the light of new Italian legislation on informed consent and to verify whether it promotes greater participation of minors in healthcare choices, given that the issue of acquisition of informed consent is becoming increasingly broad and complex.

**Conclusion:**

The case examined here indicates that current Italian legislation, even including the modifications introduced, does not allow for concrete and active participation of minors, especially those under the age of 12, in the discussion of choices about their health, not even in choices regarding the end of life, and not even when the minor manifests a mature capacity for discernment.

## 1 Introduction

Neuroblastoma is one of the most frequent extracranial tumours manifested in children under the age of 5: each year, there are 1–3 cases per 70,000 [1].

It is a malignant tumour of the cells of the embryonic neural crest, which develops into the sympathetic nervous system. Most cases of neuroblastoma are observed in the adrenal glands or the ganglia in the abdomen, while in the remaining cases it affects the ganglia along the spinal column at the level of the neck, torso or pelvis.

According to the literature, malignant forms progress along four stages [2]:

- Stage 1: localized tumour with complete gross excision, with or without microscopic residual disease; representative ipsilateral lymph nodes negative for tumour microscopically (nodes attached to and removed with the primary tumour may be positive).
- Stage 2A: localized tumour with incomplete gross excision; representative ipsilateral nonadherent lymph nodes negative for tumour microscopically.
- Stage 2B: localized tumour with or without complete gross excision, with ipsilateral nonadherent lymph nodes positive for tumour. Enlarged contralateral lymph nodes must be negative microscopically.
- Stage 3: unresectable unilateral tumour infiltrating across the midline (vertebral column) with or without regional lymph node involvement; localized unilateral tumour with contralateral regional lymph node involvement; or midline tumour with bilateral extension by infiltration (unresectable) or by lymph node involvement.
- Stage 4: any primary tumour with dissemination to distant lymph nodes, bone, bone marrow, liver, skin and/or other organs (except as defined for stage 4S).
- Stage 4S: localized primary tumour (as defined for stages 1, 2A or 2B), with dissemination limited to skin, liver and/or bone marrow (limited to infants <1 year of age).

Symptomatology is related to the location of the tumour or the metastasis; pain in the bone segment characterizes the clinical presentation of this disease, together with hyperpyrexia, sweating, flushing, tachycardia or hypertension [3].

The prognosis for children with neuroblastoma is dependent on many factors, such as age at diagnosis, disease stage and histological grade. Depending on the grade of the neuroblastoma, mortality rates can exceed 90% [4].

The present work reports the case of a 5-year-old boy diagnosed with neuroblastoma in the right adrenal loggia. The child underwent several chemotherapy treatments that prolonged his life until the age of 10.

The authors propose a 5-year clinical story that illustrates the complexity of the treatments the minor underwent to demonstrate how it can influence the minor’s condition of maturity and discernment and thus his capacity for expressing assent to or refusal of treatments.

The principle of informed consent [5] constitutes the manifestation of the freedom of self-determination of the subject in relation to his/her own health and is grounded in the Constitution (articles 2, 13, 32 Const.); besides, this principle is also seen in some articles of the Italian Medical Deontology Code [6], which view informed consent as a requisite for the lawfulness and legitimacy of every diagnostic [7] or treatment procedure.

Consent must be personal, specific, expressed, aware, informed, free and is freely revocable at any moment [8,9,10,11,12,13,14,15].

The individual’s freedom of choice includes the right to receive healthcare and request the necessary or desired treatment, as well as the subject’s negative right to autonomously choose to refuse healthcare treatments [16]. A responsible and conscious refusal constitutes an insuperable barrier for medical activity.

The Italian Constitution, Article 32, Section 2, states that no one can be obligated to accept a particular medical treatment unless it is required by law. The patient can refuse or interrupt treatment even if the choice will lead to the patient’s death. However, this freedom applies only to those of age (18 in Italy), who have acquired the “capacity to do all the actions for which a different age has not been established” (Article 2 of the Civil Code).

Given that minors juridically lack the capacity to act, they do not have free self-determination in choices about their state of health. Their wishes can only be taken into consideration by those who exercise parental responsibility, as reiterated in Law no. 219 of December 22, 2017.

Society’s expansion of spaces of “movement” and of freedom for minors poses the question of what principles should guide physicians and jurists in relation to choices to be made about the end of the life of an underage patient [17].

Is it possible to overcome the formal element of whether the patient is of age and give greater weight to the substantial aspect of what the minor perceives to be in his or her best interests? Is it possible to allow autonomy of decision to a minor below the age of 12 on a case by case basis, in relation to psychological and intellectual maturity reached through years of treatment?

His case provides the point of departure for a discussion of the legal ramifications when the child’s wishes conflict with parental decisions about treatments that can be employed in terminal oncological pathologies; the discussion does not deal with other issues that could arise, such as the attribution of parental authority [18], or possible disagreements between parents on the choices to be made.

## 2 Case presentation

The 5-year-old boy was admitted to a Marche Region (Italy) hospital with pain in the legs and in the lombo-sacral area of the spinal column. An abdominal mass was palpated by a paediatrician in the right iliac fossa. The systemic evaluations, which included routine blood and urine tests, hepatic and renal function examinations and the serum glucose test, revealed unremarkable findings, with the exception of anaemia.

Computed tomography scan highlighted multiple soft tissue mass in the right adrenal loggia. The abdominal sonography examination revealed a heterogeneous hypoechoic mass in the right adrenal gland.

Bone marrow biopsy revealed massive infiltration of neuroblasts; bone marrow aspirate did not indicate amplification of the proto-oncogene MYCN or chromosome 1p36 deletion. mIBG scintigraphy revealed hyperfixation of the radiopharmaceutical in the right adrenal gland, in the left supraclavicular region, in the lumbar spine, sternum, both humeri and both femora; further hyperaccumulations were present in the abdomen, attributed to lymphadenomegalies.

According to the clinical, radiological and histopathological findings, the patient was diagnosed with stage IV neuroblastoma.

Treatment of stage IV patients generally consists of intensive induction chemotherapy, high-dose myeloablative therapy with allogeneic or autologous bone marrow or peripheral blood stem cell transplant, surgery, radiation therapy in some cases and maintenance or biologic therapy to eradicate minimal residual disease [19].

The patient was transferred to the Department of Paediatric Oncology for the treatment of the tumour through adjunct chemotherapy according to the European NB-AR protocol [20], followed by laparoscopic surgery after 4 months. The informed consent form for medical treatment was presented to the parents, without involving the minor.

In the following months, he was given antiblastic therapy with two cycles of ifosfamide and adriamycin, followed by high doses of busulphan and melphalan. This treatment failed to eliminate the primitive lesion or the presence in the lymph nodes, and thus the induction therapy was followed by radiation therapy. Once again, the informed consent form for medical treatment was presented to the parents, without involving the minor; this was also the case for subsequent treatments.

After radiation therapy, treatment with Roaccutan (isotretinoin) was started, associated during the interval phases with Proleukin (aldesleukin) at the dose of 400,000 IU/kg for 5 days.

Ten months after the last chemotherapy treatment, there was recurrence in the proximal third of both femora and in the humeri and in the pelvis, sternum and lumbar spine. A second-level treatment with cycles of TVD (topotecan–vincristine–doxorubicin) was initiated.

After 5 months of treatment, therapy with ICE (cold therapy) began, and bone marrow aspirate was negative for neuroblastoma. This was followed by treatment with etoposide in 14-day cycles of 50 mg/day after 3 months, but another examination of the bone marrow revealed minimal neoplastic infiltration, and thus further cycles of ICE therapy were given.

When the patient was 9 years old, 4 years after diagnosis, he received allogenic bone marrow transplant at the S. Matteo Pediatric Oncology Center of Pavia. After 7 months, the bone disease recurred in the femur, pelvis and right cheekbone. The clinical conditions were poor and the marked neutropenia (WG 200 mm^3^) meant that another cycle would be inappropriate. In addition, the patient had repeated episodes of infection and so was treated with wide spectrum antibiotics.

The parents were informed of the futility of further chemotherapy, and thus the patient was discharged without any therapeutic regimen and entrusted to assisted home care and palliative treatments with complementary homeopathic therapies (sublingual drops of Synchro levels; vials of glandula suprarenalis suis-injeel Heel, vials of funiculus umbilicalis suis injeel heel, viscum album fermentatum quercum and viscum album fermentatum Pini).

The patient presented deteriorative signs at the 6-month follow-up (50/100 Karnofsky performance status scale [21]). As his neutropenia persisted, he was given blood transfusions as needed. The parents of the patient refused the recommended treatment for pain, to avoid a tendency to drowsiness. Instead, the child was given Fentanyl transdermic patch 50 μg/72 h, together with morphine per os as needed, with rotation toward metamizole sodium and indomethacin.

In response to the patient’s depression and loss of appetite, treatment with amitriptyline hydrochloride was initiated (9 drops/day).

The child asked not to be subjected to further blood transfusions and requested a stronger sedative. However, the team providing home palliative care, following the current Italian law at that time regarding parental authority, carried out the parents’ wishes for their child to continue receiving blood transfusions and a palliative care. The child died at the age of 10 years and 6 months.

According to Italian law, the authorization of an ethics committee was not necessary to describe this case report.

## 3 Discussion

This case occurred over the course of 5 years before Italian Law 201/17 [22] on informed consent came out. We thought it is interesting to examine the case in the light of the new legislation to verify whether it promotes greater participation of minors in healthcare choices, given that the issue of acquisition of informed consent is becoming increasingly broad and complex [15].

The case was doubly complicated for the physicians caring for the child, given the tender age of the patient and the conflict between his wishes and those of his parents.

Obtaining informed permission from parents or legal guardians before medical interventions on paediatric patients is now standard within our medical and legal culture [23,24]. In addition, older children and adolescents should be involved in the medical decision-making and consent process, according to the American Academy of Pediatrics (AAP) statements on informed consent [25]. By now it is well established that minors should be involved in choices regarding therapy treatments [23], clinical trials [26] and the use of biological samples for research purposes [27,28].

A child’s right to express views “in all matters affecting the child” and to have them “given due weight in accordance with the age and maturity of the child” was recognized internationally with the 1989 UN Convention on the Rights of the Child, article 12 (ratified in Italy with Law no. 176 of 27/05/1991, art. 12) [29], as well as by the 1996 European Convention of the Exercise of Children’s Rights (ratified in Italy with Law no. 77 of 20/03/03, articles 3–6) [30], the 2000 EU Charter of Fundamental Human Rights proclaimed in Nice (07/12/2000 art. 24) [31] and the 1997 Oviedo Convention on Human Rights and Biomedicine (ratified in Italy with Law no. 145 04/03/2001, art. 6) [32].

In Italy, current legislation on consent (L. 219/17 art. 3) states that “1. Minors […] have the right to the valorisation of their capacity to understand and to decide […]. The must receive information about healthcare choices in a form appropriate to their ability to understand, so that they can express their wishes…”. At the same time, it does not require that the minor’s wishes be documented, inasmuch as “2. Informed consent to medical care for the child is expressed or refused by those with parental authority or by the guardian, taking into consideration the will of the minor, in relation to his or her age and degree of maturity, with the goal of safeguarding the psychophysical health and the life of the minor, in full respect for his or her dignity” [22]. In effect, this legislation only partially considers the therapeutic relationship or alliance that must be established, first of all, between the healthcare professional and the patient, even when the patient is a minor.

Moreover, minors do not have the legal capacity to act, but, in relation to their psychological and intellectual maturity, can have full capacity of self-determination, making valid decisions. Another consideration is that while some minors may have psychological and intellectual maturity that gives them full capacity of self-determination and the ability to make valid decisions, they do not have the legal capacity to act.

According to Italian law, in art. 316 of the civil code [33], both parents have parental authority that is exercised by shared accord, keeping in mind the abilities, natural inclinations and aspirations of their child. In cases of disagreement on questions of particular importance (Art. 337 – third subsection 3 Civil Code) [33], each of the parents can turn without a formal procedure to the Judge, who, having listened to the parents and, when the child is at least 12 years old or even younger when capable of discernment, having ordered that the child be heard, suggests the solution most useful for the child and the unity of the family. If the disagreement continues, the Judge assigns decisional power to the parent who in the particular case is deemed most suitable for pursuing the interests of the child. The law does not provide indications in the case of disagreement between parents and the child. The judge can void parental authority when the parent violates or neglects responsibilities or abuses this power with grave prejudice to the child (art. 330 Civil Code) [33].

In the case examined here, both parents agreed on having blood transfusions done and providing treatment to alleviate the neoplasm-related pain but not to administer deep palliative sedation. Their son, however, requested the opposite provisions.

The traditional theory [34] requiring physicians above all to safeguard the life of the patient always and at all costs, even against the patient’s will, seems to be no longer concretely sustainable, according to interpretation of the principles evoked in Law 219/17 [22]. Current legislation sets forth the necessity for physicians to always and in all cases obtain consent for treatments and allows patients the right to refuse treatment. In effect, it legitimizes the right to die when life no longer appears worthy of being lived [35]. These considerations appear clear for patients capable of self-determination, but much more blurry regarding those who have not yet acquired juridical capacity for decision making or who are incapacitated.

According to the Medical Deontological Code [6] (CdM), the physician takes into consideration the opinion expressed by the minor in all decision-making processes that concern him or her and can report to the appropriate authorities when a treatment deemed necessary is opposed by an informed and aware minor or those with parental authority, and, in relation to the clinical conditions, can in any case proceed swiftly with the treatments held to be indispensable and undelayable. These indications should also be read in the negative, when the legal representatives of the minor impose therapeutic choices that are not appropriate to the case, crossing the threshold into therapeutic obstinacy.

According to Turri [36], the minor who refuses a medical treatment “expresses a right to resist, rather than a right to choose” and as such requires a lower level of capacity than strong and formal self-determination expressed in informed consent in the positive.

The general clause of the rights of minors establishes achievement of the best interests of the minor as the rule of behaviour, hermeneutical standard and criterion for conflict resolution in situations involving minors [36]. In the light of this clause, the minor is granted self-determination when it corresponds to the minor’s own best interests, defined by the parents, physicians and, should it come to this, the judges.

In the case examined in this study, there was a clear conflict between parental authority and the child, which shifted responsibility for guaranteeing the “good of health” of the minor and of promoting his best interests from the parents to the physician. This duty may conflict with the parent’s or patient’s wishes and set up tensions either within the family or between the family and the physician [23].

Actually, parents grant “informed permission” for the healthcare treatments with the assent of the minor, who must be informed about his or her state of health, but above all must be able to comprehend the information provided.

The capacity to comprehend derives from developmental maturity, severity of illness, educational limitations or language barriers [25], but generally this holds only in the case of older children.

The National Bioethics Committee stated in its 20/06/1992 opinion [37] that consent is conceivable for a child between 7 and 12 years, because at this age hypothetical, critical and abstract thinking about things begins, though the consent is to be conceived of together with that of the parents; with entry into adolescence there can be increasingly autonomous consent, to be considered priority after the age of 14.

In the case being examined, the child was 10 years old when he rejected further blood transfusions and requested complete elimination of his pain, possible only through recourse to deep palliative sedation.

The child’s desires were not taken into consideration by the parents, nor were they fully considered by the physicians treating him.

According to Papini, in situations of conflict, the condition of maturity and discernment of the minor should be the guiding light in the search for a suitable interpretative solution [17].

The capacity of discernment is defined as the minor’s capacity to understand what is useful for him or her and to decide autonomously from others; this entails not only cognitive but also relational ability, and above all is a specific skill gained through the dynamic relationships of the child’s family [38].

Starting with the minor’s capacity for “self-determination,” Piccinni [39] proposed three distinctions, considering

- minors who are naturally incapable: they cannot discern their own good, and thus the principal need is to protect the minor’s health;
- minors who are endowed with partial capacity: they have a minimum capacity of discernment and their will can be listened to in terms of “weak” self-determination; and
- minors who are endowed with full capacity of discernment; strong self-determination can be recognised.

It is evident that as long as articles 2 and 19 of the Civil Code stand, and the status of being of age remains the criterion for self-determination, these considerations about capacity of discernment will have no substantial value [40,41,42].

Examination of the clinical information reveals that only marginal palliative treatments were given. In contrast, Law 38/2010 [43] called for suitable and appropriate pharmacological treatments to suppress and control pain.

Considering the advanced stage of the pathology and the symptomatology, the child should have received deep palliative sedation [44], perhaps administered intermittently [45] so that the parents would have suffered less acutely the separation from their son.

Palliative sedation causes patients to be unconscious, but spares them the atrocious suffering of the final phases of a terminal illness [46]. Not only do deontological and ethical regulations [47] require that physicians administer palliative sedation, but above all, the law (art. L 291/17) directly recognizes and guarantees it as a right of the terminally ill patient.

## 4 Conclusion

The case examined here indicates that current Italian legislation, even including the modifications introduced, does not allow for concrete and active participation of minors, especially those under the age of 12, in the discussion of choices about their health, even when the minor shows good capacity of discernment.

Should the parents and the minor disagree, and should the parents refuse to accept the child’s rejection of invasive treatments and requests for a dignified and pain-free death, the role of guarantor is shifted to the physicians, who might find themselves in the position of having to involve a Tutelary Judge (art. 344 of Civil code).

Current social and medical home assistance lacks pathways for decision making and care regarding palliative sedation that respect scientific standards as well as the ethics and culture of the patient and the family. Such protocols should guarantee transparency for patients, their families, the healthcare system and society as a whole [48].

Those involved in such moral decisions and the physicians could possibly turn to an Ethics Committee [49] or request an ethics consultation [50,51] for assistance in articulating and reflecting upon their point of view, discussing it coherently, and analysing the various aspects to reach well-thought-out solutions in the best interests of the patient.

We hold that in the case of a child under the age of 12, it is necessary to take into consideration the clinical history and evaluate case by case the child’s degree of maturity, and then listen to the minor’s requests, balancing them with the child’s best interests. Medical professionals responsible for a minor should start from these conditions in planning the best treatment possible for the minor, even should it go against the will of the parents.

It is to be augured that Law 217/17 will be corrected to properly take into consideration the capacities of minors under the age of 12.
